# 

**Published:** 1884-08

**Authors:** 


### To Saratoga.

Arrangements have been made with the following railroads for reduced fare for those going to the meeting of the "American

Dental Association," held from August 5th to the 9th, viz: New York, Pennsylvania & Ohio Railroad, and New York, Lake Erie & Western Railroad Co., via Albany; also the C., C., C. & I., Lake Shore, and New York Central, via Schenectady.

By each of these the fare for round trip from Cincinnati will be $24.85. Tickets on sale from August 1st to 5th, and good for return till August 15. This is a very low rate, and certainly within the reach of every one wishing to go.

Good accommodations can be had at the less pretentious hotels and private houses for from $1.50 to $2.50 per day. Tickets can be had at the offices of the railroads here named.

### Married.

BETHEL-MORSE.—At the residence of Mr. S. B. Morse, Windsor, O., on Thursday evening, July 22, 1884, Hattie E. Morse and S. P. Bethel, D.D.S., of the class of "'84," of the Dental College, University of Michigan. May kind fortune be propitious to them.

PRIESTMAN-RENOE.—June 25, 1884, Dr. W. H. Priestman, class of '82, U. of M., to Miss Stella Renoe, of Pontiac, Ill.

Dr. P. is enjoying a good practice in Pontiac, and now, with a bright little woman to share his cares, we predict for him a pleasant sail down the "River of Time."

### CONTENTS.

### COMMUNICATIONS.

Married....... 420

---

### THE DENTAL REGISTER,

A MONTHLY MAGAZINE DEVOTED TO THE INTERESTS OF THE

**DENTAL PROFESSION.**

*Terms Reduced to* $2.00 *Per Year. Single Copies,* 25 *Cents.*

**EDITED BY PROF. J. TAFT, D.D.S.**

—AND—

**Published by SPENCER & CROCKER, Ohio Dental and Surgical Depot.**

The volume commences in January and closes in December.

The REGISTER being issued monthly is always fresh, therefore Indispensable to the practitioner who desires to keep posted up with the new inventions and improvements which are daily developed. Neither expense nor effort will be spared to give value and variety to its contents. Articles will be illustrated with well executed engravings whenever they will add to the interest or assist to explanation.

Its extensive circulation and frequency of issue make it one of the BEST DENTAL ADVERTISING MEDIUMS in the United States.

All subscriptions must begin with January or July.

Local or special notices, five lines or less, will be inserted for $3 each insertion; over five lines, 50 cts. per line.

All advertising bills payable in advance, or at furthest, after the issue of the first number containing the advertisement. All transient advertisements to be paid for in advance.

☞CONTRIBUTIONS SOLICITED.

Exclusively Editorial Communications should be addressed to the Editor.

The latest number of the REGISTER may be ordered with or without other goods at any time, and all letters should be addressed to

**SPENCER & CROCKER, Ohio Dental & Surgical Depot, Cincinnati, O.**

